# A close phylogenetic relationship between Sipuncula and Annelida evidenced from the complete mitochondrial genome sequence of *Phascolosoma esculenta*

**DOI:** 10.1186/1471-2164-10-136

**Published:** 2009-03-28

**Authors:** Xin Shen, Xiaoyin Ma, Jianfeng Ren, Fangqing Zhao

**Affiliations:** 1 Jiangsu Key Laboratory of Marine Biotechnology/College of Marine Science, Huaihai Institute of Technology, Lianyungang 222005, PR China; 2 Institute of Oceanology, Chinese Academy of Sciences, Qingdao 266071, PR China; 3 Wenzhou Medical College, Wenzhou 325035, PR China; 4 Department of Biochemistry and Molecular Biology, Pennsylvania State University, Pennsylvania 16802, USA

## Abstract

**Background:**

There are many advantages to the application of complete mitochondrial (mt) genomes in the accurate reconstruction of phylogenetic relationships in Metazoa. Although over one thousand metazoan genomes have been sequenced, the taxonomic sampling is highly biased, left with many phyla without a single representative of complete mitochondrial genome. Sipuncula (peanut worms or star worms) is a small taxon of worm-like marine organisms with an uncertain phylogenetic position. In this report, we present the mitochondrial genome sequence of *Phascolosoma esculenta*, the first complete mitochondrial genome of the phylum.

**Results:**

The mitochondrial genome of *P*.*esculenta *is 15,494 bp in length. The coding strand consists of 32.1% A, 21.5% C, 13.0% G, and 33.4% T bases (AT = 65.5%; AT skew = -0.019; GC skew = -0.248). It contains thirteen protein-coding genes (PCGs) with 3,709 codons in total, twenty-two transfer RNA genes, two ribosomal RNA genes and a non-coding AT-rich region (AT = 74.2%). All of the 37 identified genes are transcribed from the same DNA strand. Compared with the typical set of metazoan mt genomes, sipunculid lacks *trnR *but has an additional *trnM*. Maximum Likelihood and Bayesian analyses of the protein sequences show that Myzostomida, Sipuncula and Annelida (including echiurans and pogonophorans) form a monophyletic group, which supports a closer relationship between Sipuncula and Annelida than with Mollusca, Brachiopoda, and some other lophotrochozoan groups.

**Conclusion:**

This is the first report of a complete mitochondrial genome as a representative within the phylum Sipuncula. It shares many more similar features with the four known annelid and one echiuran mtDNAs. Firstly, sipunculans and annelids share quite similar gene order in the mitochondrial genome, with all 37 genes located on the same strand; secondly, phylogenetic analyses based on the concatenated protein sequences also strongly support the sipunculan + annelid clade (including echiurans and pogonophorans). Hence annelid "key-characters" including segmentation may be more labile than previously assumed.

## Background

Sipunculans (peanut worms or star worms) form a minor phylum of nonsegmented coelomate worms with bilaterally symmetrical bodies that are divisible into a trunk and a retractable introvert. In spite of low species diversity (about 150 species), sipunculans are found from tropical to Antarctic oceans [1,2]. The fossil records for sipunculans are generally rare but three species from the Lower Cambrian Maotianshan Shale were reported by Huang *et al*. (2004), suggesting that the most typical features of extant sipunculans have undergone only minor changes over the past 520 million years [3]. Although the group was first documented in 1555, their phylogenetic relations are controversial [1,4,5]. In 1767 Linnaeus described *Sipunculus nudus*, placing it within the Vermes Intestina, a group containing truly "internal worms" and other bilateral invertebrates lacking lateral appendages [6]. These were later considered to be a derived group of annelids [7]. Quatrefages (1847) proposed the name Gephyrea or "bridge group" for sipunculans, echiurans and priapulids, assuming that they represented a connection between annelids and echinoderms [8]. Hyman (1959) suggested the disposal of Gephyre on the grounds that it was simply an easy way of grouping organisms of uncertain phylogenetic affinities. Furthermore, she suggested the elevation of sipunculans to phylum status (under the name Sipunculida) [5]. Later on, Stephen (1965) proposed the name Sipuncula for the phylum [9], a term which has been widely adopted.

Scheltema (1993) maintained the presence of a molluscan-cross during cleavage as an indication to place Sipuncula as the sister taxon to the Mollusca [10]. However, cell lineage studies have shown that the concept of the molluscan-cross vs. the annelidan-cross is oversimplified and of limited phylogenetic significance [11]. Due to superficial body plan similarity, sipunculans and echiurans are often grouped together [12]. But prominent differences including anal position and proboscis form suggest that the similar body plans are a result of convergence due to parallel burrowing lifestyles, rather than common ancestry. Recently, the Echiura has been considered a derived polychaete group that may have lost segmentation [13,14], leading to a more confused placement of sipunculans.

Previous cladistic analyses based on morphological and limited molecular data have rendered a great variety of hypotheses relating Sipuncula, including sister group to Echiura [15], sister group to Annelida [16], sister group to Mollusca [10], sister group to Echiura + Annelida [17], sister group to Mollusca + Annelida [18], and sister group to an unresolved clade containing Mollusca, Annelida and the Panarthropoda [19], or within Annelida [4]. In summary, little agreement is reached with regards to the exact position of Sipuncula within the protostomes.

With a few remarkable exceptions, animal mitochondrial DNAs (mtDNAs) are circular molecules, 14–20 kb in size, containing 37 genes: 13 for proteins of electron transport (*cox1*-*3*, *cob*, *nad1*-*6*, *nad4L*, *atp6 *and *atp8*), 2 for ribosomal RNAs (*srRNA *and *lrRNA*), and 22 for transfer RNAs. Over the past decades, inference of a deeper phylogenetic relationship of Metazoa with complete mitochondrial genome sequences has gained popularity [20-22]. This resulted from many advantages offered over other molecular markers for phylogenetic analysis, such as (a) ease of isolation and assaying; (b) simple genetic structure lacking complicated features such as repetitive DNA, transposable elements, pseudogenes, and introns; and (c) effectively single copy, comparison of paralogous genes is generally not a concern [21]. In addition, mitochondrial genome provides a systematic view and measurement of evolutionary history of an organism which is synchronized with the nuclear genome of the host [23]. More importantly, compared to individual genes, mitochondrial genomes can provide sets of genome-level characteristics, such as the relative rearrangements of gene orders, which can be powerful for phylogenetic analysis [24,25].

More than one thousand complete mitochondrial genome sequences  have been reported to date. The taxonomic sampling, however, is highly biased toward vertebrates and arthropods (both groups account for ~86% of sequenced mt genomes), with no complete mt genome in many minor phyla. Minor phyla are generally considered to be of little consequence usually with uncertain affinity in mainstream animal evolution theories because they are not well represented in present macrofauna. However, if we use the questionable definition of a phylum as a taxon with a distinctly unique body plan and leave aside the requirement of monophyly, then minor phyla represent the majority of nature's experimentation with animal body plans [26].

In this paper, we described the gene content, organization and codon usage of the first complete mitochondrial genome in the phylum Sipuncula, *Phascolosoma esculenta*. We analyzed the phylogentic relationship of Sipuncula with mitochondrial genomes from Annelida, Echiura, Pogonophora, Myzostomida, Mollusca and some other protostomes. The result provides further evidence on phylogenomic scale to a close relationship of Myzostomida, Sipuncula and Annelida (including echiurans and pogonophorans).

## Results and Discussion

### General features

**Figure 1:**
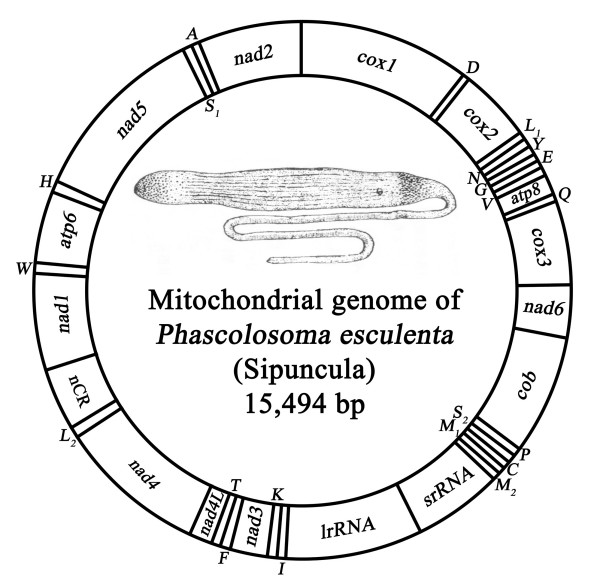
**Gene map of mitochondrial genome of the *P. esculenta *(Sipuncula: Phascolosomatidea)**. All thirteen protein-coding, two ribosomal RNA and twenty-two transfer RNA genes are encoded on the same strand. Transfer RNA genes are designated by single-letter amino acid codes.

The mitochondrial genome of *P. esculenta *is 15,494 bp in length, and encodes a set of 37 metazoan genes (thirteen protein-coding, two ribosomal RNA, and twenty-two transfer RNA genes) (Figure 1; Table 1). The overall A+T content of *P*.*esculenta *(65.5%) is higher than that of the mitochondrial genomes from the Annelida/Echiura group except for one polychaete *Clymenella torquata *(67.2%) [see additional file 1]. The entire *P*.*esculenta *mtDNA sequence has been deposited in GenBank with accession number EF583817.

**Table 1:** Mitochondrial gene profile of the *P. esculenta*

Genes and features	Positions	Size (bp)	Codon		Intergenic Nucleotides*
				
	From to		Start	Stop	
*cox1*	1–1560	1560	TTG	TAA	2
*trnD*	1563–1628	66			40
*cox2*	1669–2364	696	ATG	TAA	10
*trnL*_1_	2375–2436	62			1
*trnN*	2438–2506	69			0
*trnY*	2507–2578	72			-1
*trnE*	2578–2645	68			-3
*trnG*	2643–2708	66			3
*trnV*	2712–2774	63			13
*atp8*	2788–2952	165	ATG	TAA	6
*trnQ*	2959–3026	68			106
*cox3*	3133–3936	804	ATA	TAA	4
*nad6*	3941–4414	474	ATG	TAA	2
*cob*	4417–5552	1136	ATG	T-	0
*trnP*	5553–5617	65			-1
*trnS*_2_	5617–5683	67			-1
*trnC*	5683–5743	61			2
*trnM*_1_	5746–5807	62			0
*trnM*_2_	5808–5868	61			0
*srRNA*	5869–6706	838			0
*lrRNA*	6707–7981	1275			0
*trnI*	7982–8046	65			0
*trnK*	8047–8110	64			0
*nad3*	8111–8474	364	ATG	T-	0
*trnK*	8475–8537	63			1
*trnT*	8539–8600	62			1
*nad4L*	8602–8886	285	ATG	TAA	-4
*nad4*	8883–10233	1351	ATA	T-	0
*trnL*_2_	10234–10296	63			0
nCR	10297–10881	585			0
*nad1*	10882–11796	915	ATG	TAA	2
*trnW*	11799–11860	62			0
*atp6*	11861–12556	696	ATG	TAA	63
*trnH*	12620–12680	61			0
*nad5*	12681–14396	1716	ATG	TAA	2
*trnS*_1_	14399–14464	66			3
*trnA*	14468–14530	63			-6
*nad2*	14525–15494	970	ATG	T-	0

NOTE. * Numbers correspond to the nucleotides separating different genes.

Negative numbers indicate overlapping nucleotides between adjacent genes.

"-" indicates termination codons completed via polyadenylation.

### Gene order

**Figure 2:**
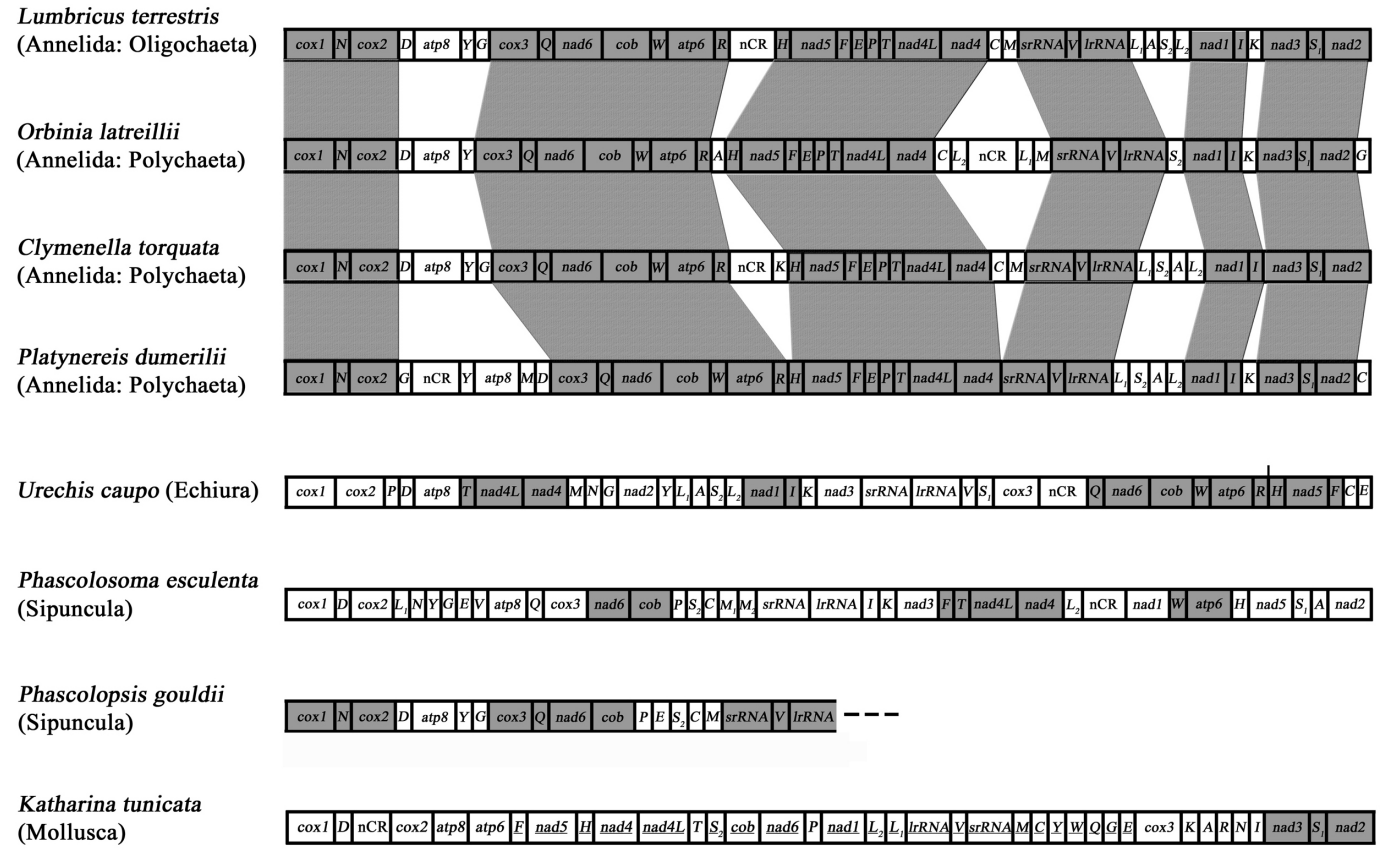
**Gene orders of mitochondrial genomes from Sipuncula and the Annelida/Echiura group**. Abbreviations are as explained in the text. Shaded boxes highlight different sets of conserved gene blocks among the taxa shown. Gene segments are not drawn to scale. All genes are transcribed from left-to-right except those indicated by underlining, which are transcribed from right to left.

All the mitochondrial genes of *P*.*esculenta *are transcribed from the same strand (Figure 1), as is the case for the four studied annelids (*Orbinia latreillii *[27], *C*.*torquata *[28], *Platynereis dumerilii *[29], and *Lumbricus terrestris *[20]), one echiuran *Urechis caupo *[30], and many other lophotrochozoan mtDNAs [31]. There may be an evolutionary "ratchet" in cases where all genes coincidentally occur on the same strand that is caused by the loss of the transcriptional signals for the opposite strand, which then makes further inversions lethal [31]. The gene synteny in *P*. *esculenta *shares moderate similarity with other four annelid mtDNAs (Figure 2).

The gene order in annelids is quite conserved with the exception of just a few tRNA genes. Comparing all the known annelid mitochondrial genomes, six conserved gene clusters can be found: (1) *cox1*-*N*-*cox2*; (2)*cox3*-*Q*-*nad6*-*cob*-*W*-*atp6*-*R*; (3) *H*-*nad5*-*F*-*E*-*P*-*T*-*nad4L*-*nad4*; (4) *srRNA*-*V*-*lrRNA*; (5) *nad1*-*I*; and (6) *nad3*-*S*_1_-*nad2 *(Figure 2). *U*.*caupo *(Echiura) contains four of them compared with annelids, including (1)*Q*-*nad6*-*cob*-*W*-*atp6*-*R*; (2)*H*-*nad5*-*F*; (3)*T*-*nad4L*-*nad4*; and (4) *nad1*-*I*. In comparison with the four annelids, three conserved gene clusters are present in *P*.*esculenta*, including (1)*nad6*-*cob*; (2)*T*-*nad4L*-*nad4*; and (3)*W*-*atp6 *(Figure 2). As to the partial mitochondrial genome of a sipunculid *Phascolopsis gouldii *[32], it also possesses three conserved regions (*cox1*-*N*-*cox2*, *cox3*-*Q*-*nad6*-*cob*, and *srRNA*-*V*-*lrRNA*), and it is surprising that the first and third gene blocks located in the *P*.*gouldii *cannot be found in the mitochondrial genomes of *U. caupo *or *P*.*esculenta *(Figure 2). Meanwhile, gene order in the mitochondrial genomes of *Riftia pachyptila *(Pogonophora) and *Myzostoma seymourcollegiorum *(Myzostomida) also showed remarkable similarities with studied annelids [28,33].

A conserved pattern of gene order across sipunculans, echiurids, pogonophorans, myzostomids, and annelids was surprising, since high variations in gene order is known to occur within closely related taxa like brachiopods [34-36] and molluscs [37-39]. Jennings and Halanych (2005) suggested that gene order data are of limited utility in Annelida [28]. On the contrary, Bleidorn *et al*. (2006) believed that such data may be a promising tool to search for synapomorphic gene rearrangements and shed light on annelid related phylogeny [27]. At this moment, it is still too early to say whether the gene order is a crucial tool or not, when complete mitochondrial genomes are still underrepresented for annelid related groups. However, from known data it can be concluded that gene rearrangements in this group may be less frequent than in other lophotrochozoan taxa, although more frequent than previously thought.

### Protein-coding genes

Mitochondrial genes commonly use several alternatives to ATG as start codons. Ten of the thirteen PCGs (*atp6*, *atp8*, *cob*, *cox2*, *nad1*-*3*, *nad4L *and *nad5*-*6*) of *P*.*esculenta *initiate with the ATG start codon, while *cox3 *and *nad4 *genes start with ATA and the *cox1 *gene with the TTG codon (Table 1). Nine open-reading frames end with the TAA stop codon (*atp6*, *atp8*, *cox1*-*3*, *nad1*, *nad4L*, *nad5 *and *nad6*), and the remaining ones (*cob*, *nad2*, *nad*3 and *nad*4) have incomplete stop codons. Such immature stop codons are common among animal mitochondrial genomes, and it has been shown that TAA stop codons are created via posttranscriptional polyadenylation [40].

Among the thirteen PCGs, there is one reading-frame overlap between *nad4L *and *nad4 *genes (Table 1) [also see additional file 2]. We speculate that *nad4L *may have an abbreviated stop codon, but is inferred to overlap with *nad4 *by four nucleotides to the first legitimate stop codon, since overlap of this pair has been commonly observed in other mtDNAs [27,30,41]. It is not clear how gene overlaps could be resolved from a polycistronic transcript, but the presence of these stop codons seems beyond coincidence. It could be that they serve as a "back up" in case translation and should begin in the absence of transcript cleavage [30].

### Base composition and codon usage

The coding strand in *P*.*esculenta *consists of 32.1% A, 21.5% C, 13.0% G, and 33.4% T bases [see additional file 3]. The bias of the base composition of an individual strand can be described by skewness [42], which measures the relative numbers of As to Ts and Gs to Cs and is calculated as (A%-T%)/(A%+T%) and (G%-C%)/(C%+G%), respectively. The PCGs have a strong skew of C vs. G (-0.191~-0.456), except that the *cox3 *gene has a weaker skew of C vs. G (-0.083); whereas the AT skew is only slightly negative for most PCGs (-0.003~-0.243) except for the *cox2 *and *atp8 *genes (AT skew = 0.146 and 0.069 in *cox2 *and *atp8 *genes, respectively). Base composition and skewness of PCGs are similar to the whole genome (AT = 65.5%; AT skew = -0.019; GC skew = -0.248) [see additional file 3]. As can be seen in the additional file 4, this is strongly reflected in the use of synonymous codons. As for two rRNA genes, GC skew is weaker than that of the whole genome (GC skew = -0.074 and -0.190 for the *srRNA *and *lrRNA *genes, respectively), which perhaps because of the requirement for base pairing in the secondary structures of the products [43]. On the contrary, the AT skew displayed an opposite pattern to the whole genome and has a slightly skew of A vs. T (AT skew = 0.068 and 0.053 for *srRNA *and *lrRNA *genes, respectively), which is consistent to four studied annelids except for *U*.*caupo *(AT skew = 0.015 for the whole genome of *U*.*caupo*) [see additional file 3].

The A+C and G+T frequency in protein-coding and ribosomal RNA genes of *P*.*esculenta*, *U*.*caupo *and the four studied annelids was calculated [see additional file 3], and the whole genome scanning of *P*.*esculenta *and *U*.*caupo *is shown in additional file 2. That the emergence of A+C is more frequent than G+T has shown in all the six mitochondrial genomes as a whole, and all thirteen PCGs and two rRNAs of *U*.*caupo *and *O*.*latreillii *displayed a similar pattern to the whole genome, which results in the highest A+C frequency of the two species. On the contrary, the remaining four species have at least one gene that G+T is more frequent than A+C [see additional file 3].

The pattern of codon usage in the *P*.*esculenta *mtDNA was also studied [see additional file 4]. There are a total of 3,709 codons in all thirteen mitochondrial PCGs, excluding incomplete termination codons. The most frequently used amino acids were Leu (17.69%), followed by Ser (9.65%), Phe (8.95%), Ile (7.74%), and Ala (7.09%). A common feature in most metazoan genomes is a bias towards a higher representation of nucleotides A and T which leads to a subsequent bias in the corresponding encoded amino acids. This result comes from the fact that the third codon positions of the PCGs in *P*.*esculenta *prefer T more than those in annelid and echiuran species. The overall AT composition of protein-coding regions is 64.9%, but at the third codon positions the AT composition elevates to 78.2%, which is higher than the average level among the mitochondrial genomes from the Annelida/Echiura group [see additional file 1].

### Transfer and Ribosomal RNA genes

**Figure 3:**
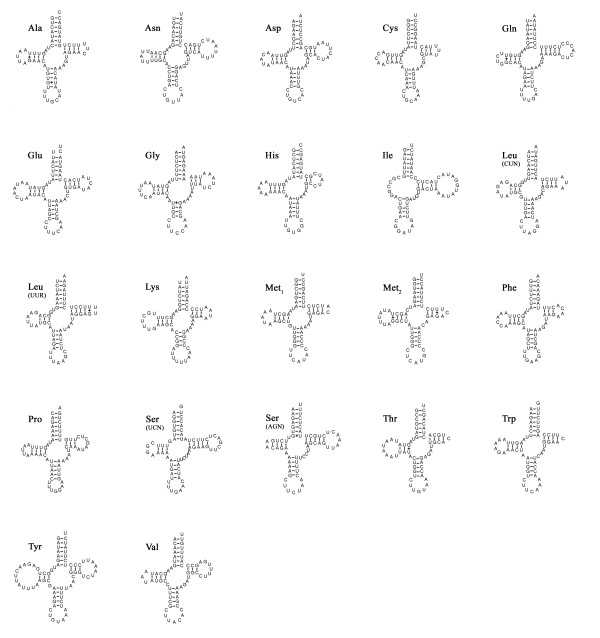
**Putative secondary structures for 22 tRNA genes of the *P. esculenta *(Sipuncula: Phascolosomatidea)**. Watson-Crick and GT bonds are denoted by "-" and "+", respectively.

The *P*.*esculenta *mtDNA encodes 22 tRNA genes, each folding into a clover-leaf secondary structure (Figure 3) and ranging in size from 61 (*trnC*, *trnM*_2 _and *trnH*) to 72 (*trnY*) nucleotides. There are five cases in total where tRNA genes appear to overlap by one to six nucleotides (Table 1). Compared with a standard set of metazoan mt genomes, the sipunculid mitochondrial genome lacks *trnR *and has an extra *trnM*. The absence of tRNA gene(s) was found in some metazoan mitochondrial genomes [44,45]. Twenty two tRNA genes were identified on the basis of their respective anticodons and secondary structures. Gene sizes and anticodon nucleotides were congruent to those described for other metazoan species.

DOGMA [46] and BLAST analyses indicate that the *srRNA *and *lrRNA *genes are adjacent to the *trnM*_2 _and *trnI *genes, and both of them are located on the coding strand. The rRNA gene boundaries were estimated from nucleotide sequence alignments with annelids species. The lengths of *srRNA *and *lrRNA *genes are 838 and 1,419 bp, and the A+T contents are 63.7% and 65.8%, respectively, which are higher than those of the mitochondrial genomes from the Annelida/Echiura group except for the *C*.*torquata *(65.4% and 70.5% for *srRNA *and *lrRNA *genes, respectively) [see additional file 1].

### Unassigned Sequence

In the mitochondrial genome of the *P*.*esculenta*, a total of 846 bp of non-coding nucleotides are scattered among eighteen intergenic regions, including a single region of 585 bp. The largest non-coding region between the *trnL*_2 _and *nad1 *is suggestive of a putative control region based on its high A+T content (AT = 74.2%) (Figure 1) [see additional file 1]. Except for the largest non-coding region, there are also three large intergenic regions adjacent to *trnQ *and *cox3*, *atp6 *and *trnH*, and *trnD *and *cox2 *(106, 63 and 40 bp in length, respectively), and others have 1 to 13 bp in length (Table 1). Tandem repeats of CAAA and TA are common in four larger intergenic regions with 16 (CAAA)s and 106 (TA)s, and an especially noteworthy (TA)_10 _was found in the largest region between the *trnL*_2 _and *nad1*.

### Phylogenomic relationship

**Figure 4:**
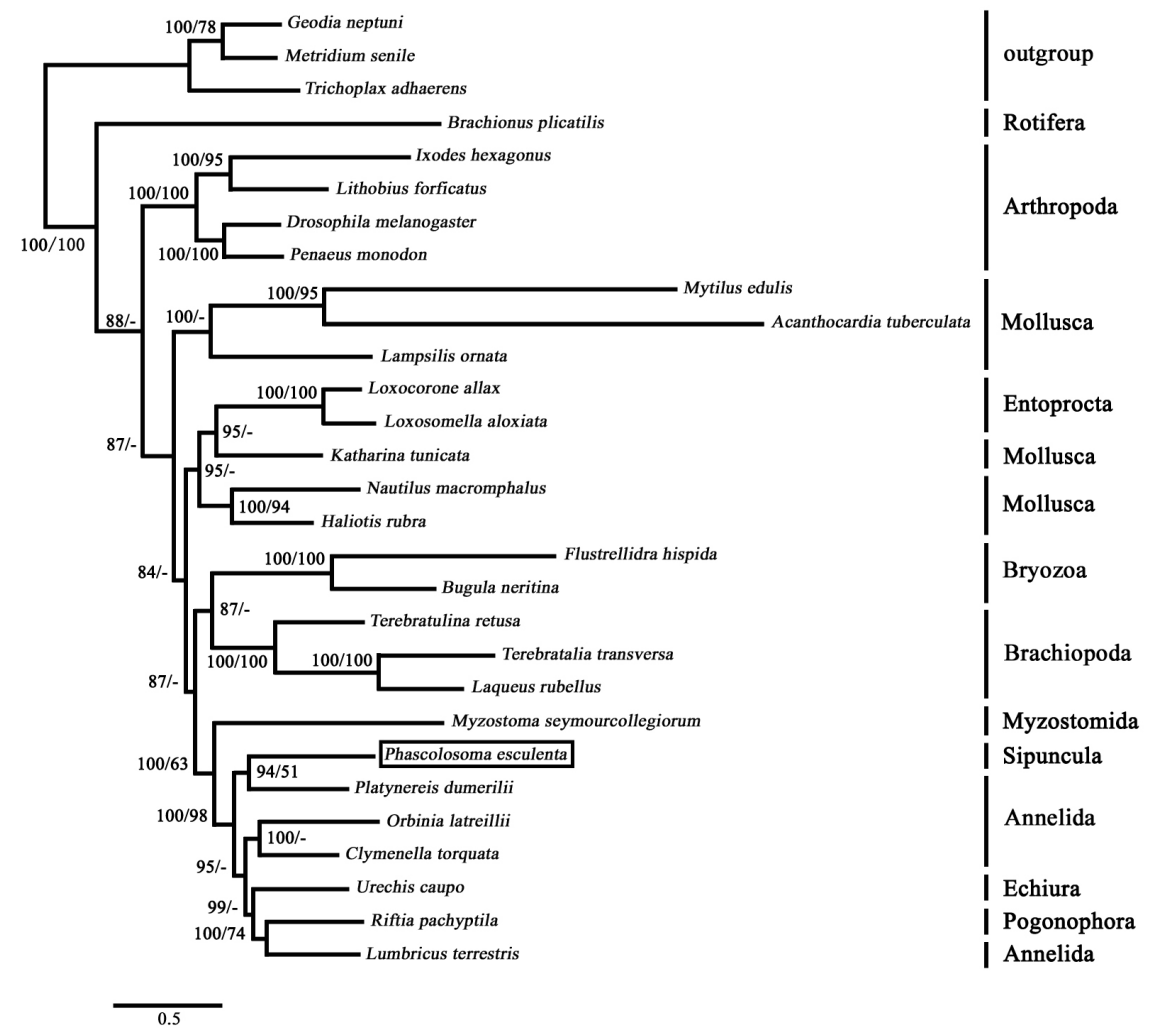
**Phylogenetic tree based on seven concatenated mitochondrial PCGs**. Tree topologies produced by the two methods were very similar. Only bootstrap values or posterior probabilities more than 50% are shown and the others are represented by "-". The first numbers are from Bayesian inferences (BPP) and the second numbers are from maximum likelihood analyses (BPM). The root of all trees was determined by using the data from Porifera, Cnidaria, and Placozoa species as the outgroup.

Phylogenies based on Maximum Likelihood (ML) and Bayesian analyses of the concatenated protein sequences were in almost complete agreement (Figure 4). In both cases, Sipuncula and Annelida (including echiurans and pogonophorans) form a monophyletic group (BPP = 100, BPM = 98), which strongly supports a closer relationship between Sipuncula and Annelida than with Mollusca, Brachiopoda, and some other lophotrochozoan groups. Gene arrangement comparisons are a powerful tool for phylogenetic studies, especially for the estimation of ancient relationships [25]. A survey of mitochondrial gene order revealed a great conservation of gene arrangements across sipunculans, annelids and echiurids [see additional file 5]. Both gene arrangement data and inferred amino acid sequences reveal that the sipunculan should be consistently and significantly clustered with annelids to the exclusion of molluscs and other taxa. Our findings are in general agreement with several published molecular studies, which grouped sipunculans with annelids closely [4,18,32,47-49]. Comparative morphological and embryological evidence provide an additional support for such relationship between Sipuncula and Annelida. Investigation of larval ocelli in pelagosphera larvae gave evidence for an annelid affinity rather than to molluscs [50]. This evidence is consistent with a morphological study of neural and muscle formation in the sipunculan *Phascolion strombus *[51]. A recent research on the neural patterning of *Phascolosoma agassizii *revealed sipunculan neurogenesis initially follows a segmental pattern similar to that of annelids, which suggests the segmental ancestry of Sipuncula [49]. If sipunculans did evolve from segmented worms, then their body plan must have changed extensively at or before the start of the Cambrian, followed by a remarkable period of stasis for the past half billion years [3]. The absence of segmentation in Sipuncula would then be a secondary loss [49], probably associated with the exploitation of a sedentary, burrowing lifestyle [52].

The hypothesis that echiurans are derived annelids was supported by our analyses (Figure 4), which is in consensus with several previous studies [13,14,53,54]. The metameric organisations of the nervous system found in *U*. *caupo *and *Bonellia viridis *are thus interpreted as an indication that echiurans are derived from a segmented ancestor [14], and the lack of segmentation in adult echiurans is therefore regarded as secondary [55]. However, the lack of segmentation in Echiura has been the single most important reason for excluding the group from the Annelida. Considering the support provided by these results for the theory that the lack of segmentation in adult echiurans is the result of reduction, it can be concluded that Echiura share the same fundamental characters that are currently regarded to constitute the bauplan of Annelida. The segmental organisation of the nervous system in combination with the numerous additional characters shared by echiurans and annelids, therefore, support a phylogenetic classification of Echiura as a subtaxon of Annelida [55].

The phylogenetic analyses based on mitochondrial genomes also confirmed the fact that pogonophorans are derived annelids (Figure 4). Pogonophorans nested within the annelids was proved by both molecular and morphological cladistic analyses [13,28,29,56]. Given the name Pogonophora is misleading at this level, McHugh proposed the name of the group reverted to that of the first family group name originally formulated for members of the group, that of Siboglinidae Caullery, 1914 [13].

The annelid affinity of myzostomids has been challenged in recent times [48]. However, Bleidorn et al. (2007) assumed that myzostomids are part of the annelid radiation based on data from nuclear genes and mitochondrial genomes [33]. Here, the phylogenetic analyses based on mitochondrial genomes confirmed a close relationship between myzostomids and sipunculans+annelids clade (including echiurans and pogonophorans) (Figure 4), which is in agreement with the morphological evidence that myzostomids are part of the annelid radiation [56,57].

Fundamentally different animal body plans, or phyla, constitute groups that are assumed to maintain their phylogenetic integrity as far back as they can be traced [13]. The great expansion of molecular data sets and improvements in phylogenetic methods have drastically changed our understanding of body plan evolution. Traditional key characteristics such as segmentation, radial versus spiral cell cleavage patterns in early embryogenesis, and modes of coelom formation appear to be more plastic and less reliable as phylogenetic characters than previously thought. Segmentation has traditionally been used as the basis for uniting annelids and arthropods as sister taxa [53], and the alternative hypothesis supports a sister relationship between molluscs and annelids, with the exclusion of arthropods, on the basis of the trochophore larva (the Ecdysozoa/Lophotrochozoa hypothesis) [58]. The placement of unsegmented Echiura and Sipuncula within Annelida radiation implies that segmentation is secondarily lost multiple times. If unsegmented echiurans are modified annelids and do not represent the unique body plan, then segmentation is an evolutionarily labile body plan character that has been lost rather than never gained by them [13].

## Conclusion

This is the first report of a complete mitochondrial genome of a representative within the phylum Sipuncula. Many features aresimilar to four studied annelids and one echiuran mtDNAs. As in case of these five and many other lophotrochozoan mtDNAs, all 37 genes are transcribed from the same DNA strand. Three conserved gene blocks compared with the four studied annelids can be identified in the *P*.*esculenta*, including (1) *nad6*-*cob*; (2) *T*-*nad4L*-*nad4*; and (3) *W*-*atp6*. Phylogenetic analyses based on inferred amino acids shown that Myzostomida, Sipuncula and Annelida (including echiurans and pogonophorans) form a monophyletic group, which supports a closer relationship between Sipuncula and Annelida than with Mollusca, Brachiopoda and some other lophotrochozoan groups. Thus, many characteristics that have been hypothesized to link sipunculans with molluscs, including their developmental pattern and lack of segmentation, should be reevaluated.

## Methods

### Sample collection and DNA extraction

Live specimen of the *P*.*esculenta *was obtained from the Wenling breeding farm (Zhejiang province, China). The muscle tissues were excised and immediately preserved at -80°C. Total genomic DNA was extracted from the tissues using a DNeasy tissue DNA extraction kit (Promega) following the manufacturer's instructions, and was dissolved in TE buffer.

### PCR and sequencing

Two partial sequences for the *cox1 *and *cox3 *genes of the *P*.*esculenta *were determined by polymerase chain reaction (PCR) using the following primer pairs: LCO1490 + HCO2198 for the fragment of *cox1 *gene and COIIIF + COIIIB for the fragment of *cox3 *gene [21].

PCR reactions were conducted in a Mastercycler gradient machine (Eppendorf AG Inc.) in a total volume of 25 μl, containing 18.0 μl sterile distilled H_2_O, 2.5 μl 10 × LA PCR buffer (Mg^2+ ^plus, Takara), 0.5 μl dNTP (10 mM each, 0.2 mM final concentration), 1 μl each primer (5 μM), 1 μl LA-Taq polymerase (1 unit, Takara), and 1 μl DNA template. The thermal cycling profile was as follows: initial denaturation at 94°C for 2 minutes and followed by denaturation at 94°C for 20 s, annealing at 52°C for 45 s, and extension at 72°C for 1 min, for 34 cycles. PCR products were purified using the Montage PCR Cleanup Kit (Millipore) and sequenced with ABI 3730x1 DNA Analyzer.

### Long PCR and sequencing by cloning and primer walking

The mitochondrial genome of *P*.*esculenta *was amplified with a long PCR protocol. Based on the partial mitochondrial genome sequences (*cox1 *and *cox3*), two pairs of primers: *cox1*-*cox3*-F (5'-AGG CTG AAC AGT CTA CCC CC-3'), *cox1*-*cox3*-R (5'-TAA TCC TAC ACA TCA CTT TGG CTT TG-3'), *cox3*-*cox1*-F (5'-AAG CCA CTC AAC ATA CCC AAA CCT AAC C-3'), *cox3*-*cox1*-R (5'-ATT GTG CTT TTC CTC ATC GTT CGT GTA G-3'), were designed to the amplification of the entire mitochondrial genome in two long PCR reactions.

PCR reactions were also done in a Mastercycler gradient machine (Eppendorf AG Inc.) and the reactions were carried out with 36 cycles of 25 μl reaction volume containing 18.5 μl sterile distilled H_2_O, 2.5 μl 10 × LA PCR buffer (Mg^2+ ^plus, Takara), 0.5 μl dNTP (10 mM each, 0.2 mM final concentration), 1 μl each primer (5 μM), 1 μl LA-Taq polymerase (1 units, Takara), and 0.5 ml DNA template. The thermal cycling profile was as follows: with an initial denaturation at 94°C for 2 minutes and followed by denaturation at 94°C for 20 s, annealing at 58°C for 45 s, and extension at 72°C for 10 minutes, for 36 cycles.

Approximately 3 μg of PCR product was sheared randomly into fragments of about 1.5 kb by forcing it repeatedly through a narrow aperture using a Hydroshear device (Gene Machines, San Carlos, CA). Following enzymatic end repair and gel purification, these fragments were ligated into pUC18 and transformed into *E*.*coli *to create plasmid libraries using standard techniques. DNA sequence data from both strands was generated from single clones using the primer walking approach, which also conducted with ABI 3730x1 DNA Analyzer.

### Sequence analysis

Base calling was performed with phred [59,60] and sequence reads were assembled in phrap with default parameters. All assembled sequences were manually checked by CONSED to remove misassemblies [61]. The locations of the 13 PCGs and two rRNAs were determined with DOGMA [46], and subsequent alignments with *C. torquata*, *L. terrestris *and *O. latreillii *(Annelida). The majority of tRNA genes were identified by using tRNAscan-SE 1.21 [62], employing the default search mode and the invertebrate mitochondrial genetic code for tRNA structure prediction. Remaining tRNA genes were identified by inspecting sequences for tRNA-like secondary structures and anticodons.

### Phylogenomic analysis

Besides the mitochondrial genome of *P*.*esculenta*, partial or complete mitochondrial genomes from Annelida, Echiura, Pogonophora, Myzostomida, Brachiopoda, Ectoprocta, Bryozoa, and Rotifera were included in the phylogenetic analysis. Six genomes from four classes of Mollusca and four genomes from four Arthropoda major clades were also included. The root of all trees was determined by using the data from Porifera, Cnidaria, and Placozoa species as the outgroup [see additional file 6].

Partial mitochondrial genome sequences of *R*.*pachyptila *(Pogonophora) and *M*.*seymourcollegiorum *(Myzostomida) contain 11 PCGs (*cox1*, *cox2*, *cox3*, *cob*, *nad1*, *nad2*, *nad3*, *nad4*, *nad6*, *atp6 *and *atp8*) and 10 PCGs (*cox1*, *cox2*, *cox3*, *cob*, *nad4*, *nad4L*, *nad5*, *nad6*, *atp6 *and *atp8*), respectively. In addition, *atp8 *gene is missing in some mitochondrial genomes. Thus the amino acid sequences from seven shared PCGs (*cox1*, *cox2*, *cox3*, *cob*, *nad4*, *nad6 *and *atp6*) were aligned using Clustal X with the default settings [63]. The final alignment for the 29 taxa consisted of 2,718 sites. Two phylogenetic reconstruction approaches was applied including Maximum Likelihood (ML) using PhyML 3.0 [64] and Bayesian inference analyses using MrBayes 3.1 MPI version [65].

Model selection for the amino acid dataset was done with ProtTest [66]. For a likelihood analysis, we implemented the MtArt matrix in PhyML 3.0 [64]. As the MtArt model is a very recent addition to the models commonly used [67], we could not implement it in Bayesian analysis, where we used the best scoring alternative, MtRev matrix and the *gamma*+*invar *model of evolutionary change. In the ML method, the assessment of node reliability was done using 1,000 bootstrap replicates (BPM). In the case of the Bayesian analyses, the Markov Chain Monte Carlo analyses were run for 1,000,000 generations (sampling every 100 generations) to allow adequate time for convergence. After approximate 100,000 generations, the log-likelihood values of each sampled tree had stabilized. After omitting the first 1,000 "burn in" trees, the remaining 9,000 sampled trees were used to estimate the 50% majority rule consensus tree and the Bayesian posterior probabilities (BPP).

## Abbreviations

*atp6*, and *8*: ATPase subunits 6 and 8; bp: base pair (s); *cox1-3*: cytochrome c oxidase subunits I-III; PCGs: protein-coding genes; nCR: non coding region; *cob*: cytochrome b; mtDNA: mitochondrial DNA; *nad1-6*, and *4L*: NADH dehydrogenase subunits 1–6 and 4L; *srRNA*, and *lrRNA*: small and large subunits ribosomal RNA; tRNA: transfer RNA; *L*_1_: *tRNA*^*Leu*(*CUN*)^; *L*_2_: *tRNA*^*Leu*(*UUR*)^; *S*_1_: *tRNA*^*Ser*(*AGN*)^; *S*_2_: *tRNA*^*Ser*(*UCN*)^.

## Authors' contributions

XS designed this study, performed all of the phylogenetic analyses, interpreted data and wrote the manuscript. XM and JR did the majority of the laboratory work and the primary sequence analysis. FZ was responsible for bioinformatic analyses. All authors read and approved the final manuscript.

## Supplementary Material

Additional file 1 Genomic characteristics of six mitochondrial genomes. Genomic characteristics of the mitochondrial genomes of *Phascolosoma esculenta *(Sipuncula), *Urechis caupo *(Echiura) and four annelids (*Orbinia latreillii*, *Clymenella torquata*, *Platynereis dumerilii*, and *Lumbricus terrestris*). Click here for file

Additional file 2 A+C and G+T composition along mt genomes of *Phascolosoma esculenta *and *Urechis caupo*. Plot of A+C and G+T composition along mt genomes of *Phascolosoma esculenta *and *Urechis caupo *using a sliding window of 100 nucleotides. The scaled gene maps are also presented and *tRNA *genes are pictured but not labelled. Click here for file

Additional file 3 The base composition and skew in the mitochondrial protein-coding and ribosomal RNA genes. The AT, GC skew and AC, GT frequency in the mitochondrial protein-coding and ribosomal RNA genes of *Phascolosoma esculenta *(Sipuncula), *Urechis caupo *(Echiura) and four annelids (*Orbinia latreillii*, *Clymenella torquata*, *Platynereis dumerilii*, and *Lumbricus terrestris*). Click here for file

Additional file 4 Codon usage in 13 mitochondrial PCGs. Codon usage in 13 mitochondrial PCGs of the *Phascolosoma esculenta *(Sipuncula: Phascolosomatidea). Click here for file

Additional file 5 Gene arrangements in 19 taxa. Table of gene arrangements in 19 taxa. Those matching annelids conserved gene blocks are shown in red colour. Click here for file

Additional file 6 Mitochondrial genomes used for the phylogenetic reconstruction. The list of mitochondrial genomes used for the phylogenetic reconstruction. Click here for file
