# Supplementary material for: A close phylogenetic relationship between Sipuncula and Annelida evidenced from the complete mitochondrial genome sequence of Phascolosoma esculenta
Source: BMC Genomics. 2009 Mar 28;10:136. doi: 10.1186/1471-2164-10-136 (PMC2667193; doi:10.1186/1471-2164-10-136)
Supplement: Additional file 1 — Genomic characteristics of six mitochondrial genomes. Genomic characteristics of the mitochondrial genomes of Phascolosoma esculenta (Sipuncula), Urechis caupo (Echiura) and four annelids (Orbinia latreillii, Clymenella torquata, Platynereis dumerilii, and Lumbricus terrestris). [file 1471-2164-10-136-S1.doc]

| Species | GenBank | Coding-strand | | 13 Protein-coding genes | | | | | *lrRNA* gene | | *srRNA* gene | | *tRNA* genes | | Putative control region | | Reference |
| --- | --- | --- | --- | --- | --- | --- | --- | --- | --- | --- | --- | --- | --- | --- | --- | --- | --- |
|  | Accession | Length | A+T | No. of | A+T (%) | | | | Length | A+T | Length | A+T | Length | A+T | Length | A+T |  |
|  | No. | (bp) | (%) | AA | All Positions | First Codon Positions | Second Codon Positions | Third Codon Positions | (bp) | (%) | (bp) | (%) | (bp) | (%) | (bp) | (%) |  |
| *Phascolosoma esculenta* | EF583817 | 15494 | 65.5 | 3700 | 64.9 | 55.8 | 60.5 | 78.2 | 1275 | 67.5 | 838 | 63.7 | 1419 | 65.8 | 585 | 74.2 | This study |
| *Clymenella torquata* | NC_006321 | 15538 | 67.2 | 3707 | 66.1 | 59.6 | 61.9 | 76.9 | 1295 | 70.5 | 821 | 65.4 | 1426 | 68 | 822 | 76.5 | [1] |
| *Orbinia latreillii* | NC_007933 | 15558 | 61.1 | 3685 | 60.2 | 53.1 | 59.8 | 67.9 | 1334 | 62.4 | 833 | 58.9 | 1461 | 61.7 | 633 | 74.1 | [2] |
| *Platynereis dumerilii* | NC_000931 | 15619 | 64.1 | 3661 | 63.1 | 58.9 | 60.7 | 69.6 | 1172 | 64.3 | 790 | 63.2 | 1397 | 64.6 | 1091 | 71.9 | [3] |
| *Lumbricus terrestris* | NC_001673 | 14998 | 61.6 | 3702 | 60.9 | 56.7 | 60.6 | 65.4 | 1245 | 65 | 785 | 59.6 | 1395 | 64.5 | 384 | 64.3 | [4] |
| *Urechis caupo* | NC_006379 | 15113 | 62.0 | 3710 | 61.3 | 54.1 | 59.9 | 70.0 | 1266 | 62.8 | 903 | 62.9 | 1428 | 64.4 | 282 | 71.0 | [5] |

1. Jennings RM, Halanych KM: **Mitochondrial genomes of *Clymenella torquata* (Maldanidae) and *Riftia pachyptila* (Siboglinidae): evidence for conserved gene order in annelida**. *Molecular biology and evolution* 2005, **22**(2):210-222.

2. Bleidorn C, Podsiadlowski L, Bartolomaeus T: **The complete mitochondrial genome of the orbiniid polychaete *Orbinia latreillii* (Annelida, Orbiniidae)--A novel gene order for Annelida and implications for annelid phylogeny**. *Gene* 2006, **370**:96-103.

3. Boore JL, Brown WM: **Mitochondrial genomes of Galathealinum, Helobdella, and Platynereis: sequence and gene arrangement comparisons indicate that Pogonophora is not a phylum and Annelida and Arthropoda are not sister taxa**. *Molecular biology and evolution* 2000, **17**(1):87-106.

4. Boore JL, Brown WM: **Complete sequence of the mitochondrial DNA of the annelid worm *Lumbricus terrestris***. *Genetics* 1995, **141**(1):305-319.

5. Boore JL: **Complete mitochondrial genome sequence of *Urechis caupo*, a representative of the phylum Echiura**. *BMC genomics* 2004, **5**(1):67.
