# Supplementary figures and images for: A close phylogenetic relationship between Sipuncula and Annelida evidenced from the complete mitochondrial genome sequence of Phascolosoma esculenta
Source: BMC Genomics. 2009 Mar 28;10:136. doi: 10.1186/1471-2164-10-136 (PMC2667193; doi:10.1186/1471-2164-10-136)

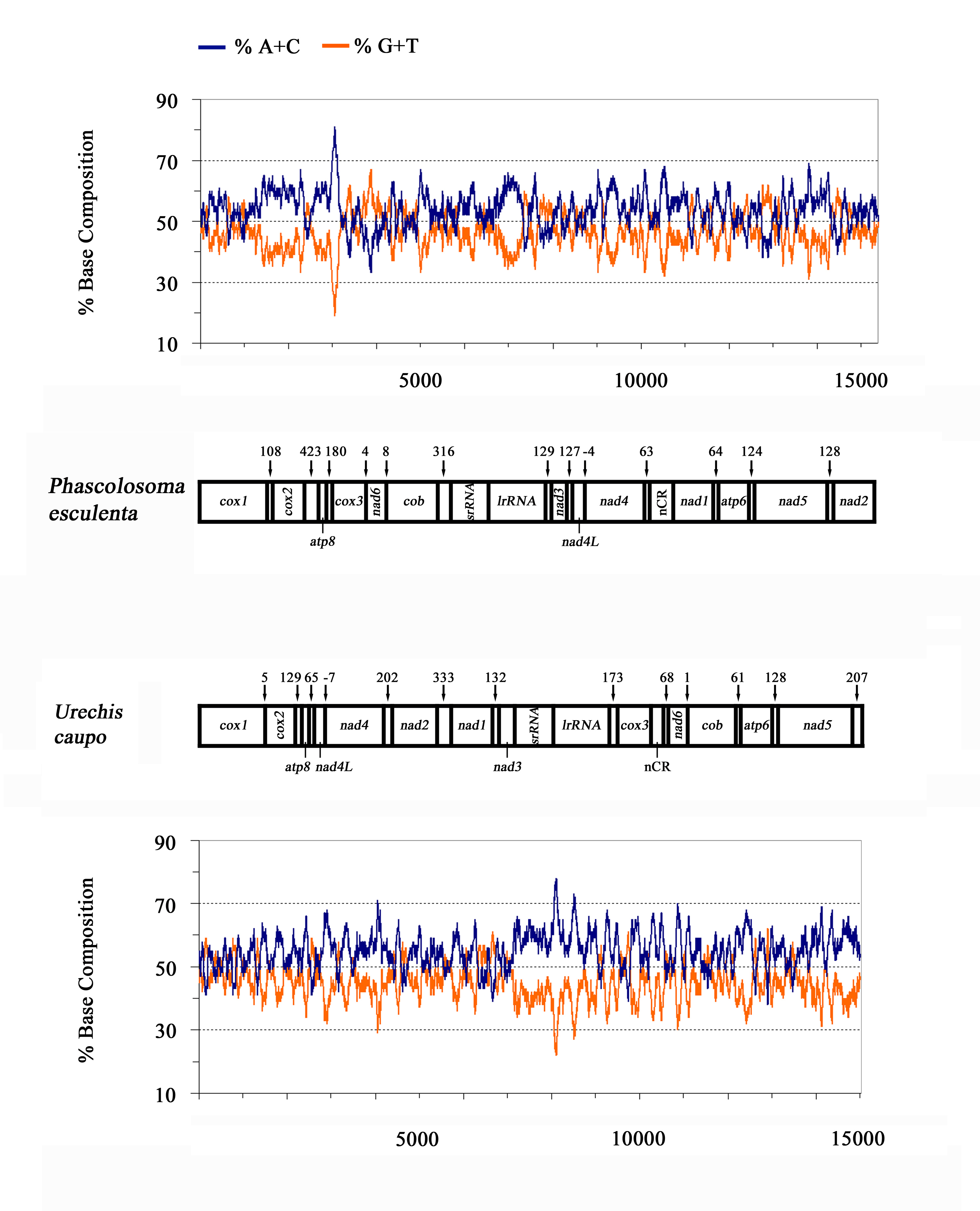

Supplement: Additional file 2 — A+C and G+T composition along mt genomes of Phascolosoma esculenta and Urechis caupo. Plot of A+C and G+T composition along mt genomes of Phascolosoma esculenta and Urechis caupo using a sliding window of 100 nucleotides. The scaled gene maps are also presented and tRNA genes are pictured but not labelled. [file 1471-2164-10-136-S2.jpeg]
