# Supplementary material for: A close phylogenetic relationship between Sipuncula and Annelida evidenced from the complete mitochondrial genome sequence of Phascolosoma esculenta
Source: BMC Genomics. 2009 Mar 28;10:136. doi: 10.1186/1471-2164-10-136 (PMC2667193; doi:10.1186/1471-2164-10-136)
Supplement: Additional file 4 — Codon usage in 13 mitochondrial PCGs. Codon usage in 13 mitochondrial PCGs of the Phascolosoma esculenta (Sipuncula: Phascolosomatidea). [file 1471-2164-10-136-S4.doc]

| AA | Codon | *N* | % | AA | Codon | *N* | % | AA | Codon | *N* | % | AA | Codon | *N* | % |
| --- | --- | --- | --- | --- | --- | --- | --- | --- | --- | --- | --- | --- | --- | --- | --- |
| **Phe** | TTT | 214 | 5.77 | **Ser** | TCT | 72 | 1.94 | **Tyr** | TAT | 61 | 1.64 | **Cys** | TGT | 20 | 0.54 |
|  | TTC | 118 | 3.18 |  | TCC | 47 | 1.27 |  | TAC | 51 | 1.38 |  | TGC | 15 | 0.40 |
| **Leu** | TTA | 198 | 5.34 |  | TCA | 120 | 3.24 | **Term** | TAA | 9 | 0.24 | **Trp** | TGA | 96 | 2.59 |
|  | TTG | 9 | 0.24 |  | TCG | 3 | 0.08 |  | TAG | 0 | 0.00 |  | TGG | 11 | 0.30 |
|  |  |  |  |  |  |  |  |  |  |  |  |  |  |  |  |
| **Leu** | CTT | 139 | 3.75 | **Pro** | CCT | 42 | 1.13 | **His** | CAT | 38 | 1.02 | **Arg** | CGT | 8 | 0.22 |
|  | CTC | 52 | 1.40 |  | CCC | 13 | 0.35 |  | CAC | 45 | 1.21 |  | CGC | 9 | 0.24 |
|  | CTA | 247 | 6.66 |  | CCA | 129 | 3.48 | **Gln** | CAA | 69 | 1.86 |  | CGA | 47 | 1.27 |
|  | CTG | 11 | 0.30 |  | CCG | 7 | 0.19 |  | CAG | 7 | 0.19 |  | CGG | 1 | 0.03 |
|  |  |  |  |  |  |  |  |  |  |  |  |  |  |  |  |
| **Ile** | ATT | 199 | 5.37 | **Thr** | ACT | 61 | 1.64 | **Asn** | AAT | 59 | 1.59 | **Ser** | AGT | 16 | 0.43 |
|  | ATC | 88 | 2.37 |  | ACC | 40 | 1.08 |  | AAC | 58 | 1.56 |  | AGC | 26 | 0.70 |
| **Met** | ATA | 173 | 4.66 |  | ACA | 134 | 3.61 | **Lys** | AAA | 80 | 2.16 |  | AGA | 70 | 1.89 |
|  | ATG | 14 | 0.38 |  | ACG | 2 | 0.05 |  | AAG | 6 | 0.16 |  | AGG | 4 | 0.11 |
|  |  |  |  |  |  |  |  |  |  |  |  |  |  |  |  |
| **Val** | GTT | 61 | 1.64 | **Ala** | GCT | 72 | 1.94 | **Asp** | GAT | 22 | 0.59 | **Gly** | GGT | 39 | 1.05 |
|  | GTC | 15 | 0.40 |  | GCC | 66 | 1.78 |  | GAC | 35 | 0.94 |  | GGC | 33 | 0.89 |
|  | GTA | 94 | 2.53 |  | GCA | 124 | 3.34 | **Glu** | GAA | 72 | 1.94 |  | GGA | 119 | 3.21 |
|  | GTG | 3 | 0.08 |  | GCG | 1 | 0.03 |  | GAG | 1 | 0.03 |  | GGG | 14 | 0.38 |

**NOTE.** AA = amino acid; *N* = total number of particular codon in all proteins; Term = termination codons. The total number of codons was 3,709. Incomplete termination codons were excluded.
