# Supplementary material for: A close phylogenetic relationship between Sipuncula and Annelida evidenced from the complete mitochondrial genome sequence of Phascolosoma esculenta
Source: BMC Genomics. 2009 Mar 28;10:136. doi: 10.1186/1471-2164-10-136 (PMC2667193; doi:10.1186/1471-2164-10-136)
Supplement: Additional file 5 — Gene arrangements in 19 taxa. Table of gene arrangements in 19 taxa. Those matching annelids conserved gene blocks are shown in red colour. [file 1471-2164-10-136-S5.doc]

| **Species** | **Clade** | **Gene arrangement** |
| --- | --- | --- |
| *Phascolosoma esculenta* | Sipuncula | cox1, D, cox2, L1, N, Y, G, E, V, atp8, Q, cox3, nad6, cob, P, S2, C, M, M, srRNA, lrRNA, I, K, nad3, F, T, nad4L, nad4, L2, nCR, nad1, W, atp6, H, nad5, S1, A, nad2 |
| *Clymenella torquata* | Annelida, Polychaeta, Scolecida, Maldanidae | cox1, N, cox2, D, atp8, Y, G, cox3, Q, nad6, cob, W, atp6, R, nCR, K, H, nad5, F, E, P, T, nad4L, nad4, C, M, srRNA, V, lrRNA, L1, S2, A, L2, nad1, I, nad3, S1, nad2 |
| *Orbinia latreillii* | Annelida, Polychaeta, Scolecida, Orbiniidae | cox1, N, cox2, D, atp8, Y, cox3, Q, nad6, cob, W, atp6, R, A, H, nad5, F, E, P, T, nad4L, nad4, C, L2, nCR, L1, M, srRNA, V, lrRNA, S2, nad1, I, K, nad3, S1, nad2, G |
| *Platynereis dumerilii* | Annelida, Polychaeta, Palpata | cox1, N, cox2, G, nCR, Y, atp8, M, D, cox3, Q, nad6, cob, W, atp6, R, H, nad5, F, E, P, T, nad4L, nad4, srRNA, V, lrRNA, L1, S2, A, L2, nad1, I, K, nad3, S1, nad2, C |
| *Lumbricus terrestris* | Annelida, Clitellata, Oligochaeta | cox1, N, cox2, D, atp8, Y, G, cox3, Q, nad6, cob, W, atp6, R, nCR, H, nad5, F, E, P, T, nad4L, nad4, C, M, srRNA, V, lrRNA, L1, A, S2, L2, nad1, I, K, nad3, S1, nad2 |
| *Urechis caupo* | Echiura | cox1, cox2, P, D, atp8, T, nad4L, nad4, M, N, G, nad2, Y, L1, A, S2, L2, nad1, I, K, nad3, srRNA, lrRNA, V, S1, cox3, nCR, Q, nad6, cob, W, atp6, R, H, nad5, F, C, E |
| *Terebratulina retusa* | Brachiopoda, Articulata | cox1, cox2, D, atp8, atp6, Y, C, M, srRNA, V, lrRNA, L1, A, L2, nad1, nad6, P, cob, K, N, S2, nad4L, nad4, Q, W, H, nad5, F, E, G, cox3, T, R, I, nad3, S1, nad2 |
| *Laqueus rubellus* | Brachiopoda, Articulata | cox1, V, cob, H, atp6, Q, W, nad5, A, nad6, I, nad3, T, R, F, E, K, S2, nad4L, G, cox3, D, atp8, S1, nad2, cox2, L1, lrRNA, srRNA, M, L2, P, nad1, Y, nad4, C, N |
| *Terebratalia transversa* | Brachiopoda, Articulata | cox1, C, cob, G, atp8, cox3, H, atp6, Q, W, nad5, L2, P, D, srRNA, M, A, nad6, L1, lrRNA, E, N, nad1, V, Y, nad4, F, S1, nad4L, cox2, I, nad3, T, R, K, S1, nad2 |
| *Lampsilis ornata* | Mollusca, Bivalvia | cox1, cox3, atp6, D, atp8, nad4L, nad4, -nad6, -G, -nad1,-L2, -V, -I, -C, -Q, nad5, -F, -cob, -P, -N,-L1, -lrRNA, -Y, -T, -K, -srRNA,-R, -W, -M, nCR, -nad2, -E, -S1, -S2, -A, H, nad3, cox2 |
| *Katharina tunicata* | Mollusca, Polyplacophora | cox1, D, nCR, cox2, atp8, atp6, -F, -nad5, -H, -nad4, -nad4L, T, -S2, -cob, -nad6, P, -nad1, -L2, -L1, -lrRNA, -V, -srRNA, -M, -C, -Y, -W, -Q, -G, -E, cox3, K, A, R, N, I, nad3, S1, nad2 |
| *Haliotis rubra* | Mollusca, Gastropoda | cox1, cox2, atp8, atp6, -F, -nad5, -H, -nad4, nad4L, T, -S2, -cob, -nad6, -P, -nad1, -L2, -L1, -lrRNA, -V, -srRNA, -M, -Y, -C, -W, -Q, -G, -E, nCR, cox3, D, K, A, R, I, nad3, N, S1, nad2 |
| *Nautilus macromphalus* | Mollusca, Cephalopoda | cox1, cox2, D, atp8, -F, -L2, -L1, -lrRNA, -V, -srRNA, -M, -C, -Y, -W, -Q, nCR, T, -G, atp6, -nad5, -H, -nad4, -nad4L, -S2, -cob, -nad6, -P, -nad1, -E, cox3, A, R, N, I, nad3, S1, nad2 |
| *Acanthocardia tuberculata* | Mollusca, Bivalvia | cox1, P, nad4L, L2, nad6, cox2, D, M, nCR, H, nad3, M,W, K, L1, nad1, F, srRNA, Q, R, I, cox3, S1, T, nad5, C, S2, cob, lrRNA, N, nad4, Y, atp6, E, G, V, nad2, A. |
| *Mytilus edulis* | Mollusca, Bivalvia | cox1, atp6, T, nad4L, nad5, nad6, F, srRNA, G, N, E, C, I, Q, D, lrRNA, nCR, Y, cob, cox2, K, M, L1, L2, nad1, V, nad4, cox3, S2, M, nad2, R, W, A, S1, H, P, nad3 |
| *Drosophila melanogaster* | Arthropoda, Hexapoda | cox1, L2, cox2, K, D, atp8, atp6, cox3, G, nad3, A, R, N, S1, E, -F, -nad5, -H, -nad4, -nad4L, T, -P, nad6, cob, S2, -nad1, -L1, -lrRNA, -V, -srRNA, I, -Q, M, nad2, W, -C, -Y |
| *Penaeus monodon* | Arthropoda, Crustacea | cox1, L2, cox2, K, D, atp8, atp6, cox3, G, nad3, A, R, N, S1, E, -F, -nad5, -H, -nad4, -nad4L, T, -P, nad6, cob, S2, -nad1, -L1, -lrRNA, -V, -srRNA, I, -Q, M, nad2, W, -C, -Y |
| *Ixodes hexagonus* | Arthropoda, Chelicerata | cox1, cox2, K, D, atp8, atp6, cox3, G, nad3, A, R, N, S1, E, -F, -nad5, -H, -nad4, -nad4L, T, -P, nad6, cob, S2, -nad1, -L2, -L1, -lrRNA, -V, -srRNA, nCR, I, -Q, M, nad2, W, -C, -Y |
| *Lithobius forficatus* | Arthropoda, Myriapoda | cox1, cox2, K, D, atp8, atp6, cox3, G, nad3, A, R, N, S1, E, -F, -nad5, -H, -nad4, -nad4L, T, -P, nad6, cob, S2, -nad1, -L2, -L1, -lrRNA, -V, -srRNA, nCR, -C, I, -Q, M, nad2, W, -Y |
