# Supplementary material for: A close phylogenetic relationship between Sipuncula and Annelida evidenced from the complete mitochondrial genome sequence of Phascolosoma esculenta
Source: BMC Genomics. 2009 Mar 28;10:136. doi: 10.1186/1471-2164-10-136 (PMC2667193; doi:10.1186/1471-2164-10-136)
Supplement: Additional file 6 — Mitochondrial genomes used for the phylogenetic reconstruction. The list of mitochondrial genomes used for the phylogenetic reconstruction. [file 1471-2164-10-136-S6.doc]

| Species | Clade | GenBank Number | Reference |
| --- | --- | --- | --- |
| *Phascolosoma esculenta* | Sipuncula | EF583817 | This study |
| *Clymenella torquata* | Annelida, Polychaeta, Scolecida, Maldanidae | NC_006321 | [1] |
| *Orbinia latreillii* | Annelida, Polychaeta, Scolecida, Orbiniidae | NC_007933 | [2] |
| *Platynereis dumerilii* | Annelida, Polychaeta, Palpata | NC_000931 | [3] |
| *Lumbricus terrestris* | Annelida, Clitellata, Oligochaeta | NC_001673 | [4] |
| *Urechis caupo* | Echiura | NC_006379 | [5] |
| *Loxocorone allax* | Entoprocta | NC_010431 | [6] |
| *Loxosomella aloxiata* | Entoprocta | NC_010432 | [6] |
| *Bugula neritina* | Bryozoa | NC_010197 | † |
| *Flustrellidra hispida* | Bryozoa | NC_008192 | [7] |
| *Brachionus plicatilis* | Rotifera | NC_010472  NC_010484 | [8] |
| *Riftia pachyptila* | Pogonophora | AY741662 | [1] |
| *Myzostoma seymourcollegiorum* | Myzostomida | EF506562 | [9] |
| *Terebratulina retusa* | Brachiopoda, Articulata | NC_000941 | [10] |
| *Laqueus rubellus* | Brachiopoda, Articulata | NC_002322 | [11] |
| *Terebratalia transversa* | Brachiopoda, Articulata | NC_003086 | [12] |
| *Lampsilis ornata* | Mollusca, Bivalvia | NC_005335 | [13] |
| *Katharina tunicata* | Mollusca, Polyplacophora | NC_001636 | [14] |
| *Haliotis rubra* | Mollusca, Gastropoda | NC_005940 | [15] |
| *Nautilus macromphalus* | Mollusca, Cephalopoda | NC_007980 | [16] |
| *Acanthocardia tuberculata* | Mollusca, Bivalvia | NC_008452 | [17] |
| *Mytilus edulis* | Mollusca, Bivalvia | NC_006161 | [18] |
| *Drosophila melanogaster* | Arthropoda, Hexapoda | NC_001709 | [19] |
| *Penaeus monodon* | Arthropoda, Crustacea | NC_002184 | [20] |
| *Ixodes hexagonus* | Arthropoda, Chelicerata | NC_002010 | [21] |
| *Lithobius forficatus* | Arthropoda, Myriapoda | NC_002629 | [22] |
| Outgroup |  |  |  |
| *Geodia neptuni* | Porifera | NC_006990 | [23] |
| *Metridium senile* | Cnidaria | NC_000933 | [24] |
| *Trichoplax adhaerens* | Placozoa | NC_008151 | [25] |

Note: † unpublished

1. Jennings RM, Halanych KM: **Mitochondrial genomes of *Clymenella torquata* (Maldanidae) and *Riftia pachyptila* (Siboglinidae): evidence for conserved gene order in annelida**. *Mol Biol Evol* 2005, **22**(2):210-222.

2. Bleidorn C, Podsiadlowski L, Bartolomaeus T: **The complete mitochondrial genome of the orbiniid polychaete *Orbinia latreillii* (Annelida, Orbiniidae)--A novel gene order for Annelida and implications for annelid phylogeny**. *Gene* 2006, **370**:96-103.

3. Boore JL, Brown WM: **Mitochondrial genomes of Galathealinum, Helobdella, and Platynereis: sequence and gene arrangement comparisons indicate that Pogonophora is not a phylum and Annelida and Arthropoda are not sister taxa**. *Mol Biol Evol* 2000, **17**(1):87-106.

4. Boore JL, Brown WM: **Complete sequence of the mitochondrial DNA of the annelid worm *Lumbricus terrestris***. *Genetics* 1995, **141**(1):305-319.

5. Boore JL: **Complete mitochondrial genome sequence of *Urechis caupo*, a representative of the phylum Echiura**. *BMC Genomics* 2004, **5**(1):67.

6. Yokobori S, Iseto T, Asakawa S, Sasaki T, Shimizu N, Yamagishi A, Oshima T, Hirose E: **Complete nucleotide sequences of mitochondrial genomes of two solitary entoprocts, Loxocorone allax and Loxosomella aloxiata: implications for lophotrochozoan phylogeny**. *Mol Phylogenet Evol* 2008, **47**(2):612-628.

7. Waeschenbach A, Telford MJ, Porter JS, Littlewood DT: **The complete mitochondrial genome of Flustrellidra hispida and the phylogenetic position of Bryozoa among the Metazoa**. *Mol Phylogenet Evol* 2006, **40**(1):195-207.

8. Suga K, Mark Welch DB, Tanaka Y, Sakakura Y, Hagiwara A: **Two circular chromosomes of unequal copy number make up the mitochondrial genome of the rotifer Brachionus plicatilis**. *Mol Biol Evol* 2008, **25**(6):1129-1137.

9. Bleidorn C, Eeckhaut I, Podsiadlowski L, Schult N, McHugh D, Halanych KM, Milinkovitch MC, Tiedemann R: **Mitochondrial genome and nuclear sequence data support myzostomida as part of the annelid radiation**. *Mol Biol Evol* 2007, **24**(8):1690-1701.

10. Stechmann A, Schlegel M: **Analysis of the complete mitochondrial DNA sequence of the brachiopod *Terebratulina retusa* places Brachiopoda within the protostomes**. *Proc R Soc Lond B Biol Sci* 1999, **266**(1433):2043-2043.

11. Noguchi Y, Endo K, Tajima F, Ueshima R: **The mitochondrial genome of the brachiopod *Laqueus rubellus***. *Genetics* 2000, **155**(1):245-259.

12. Helfenbein KG, Brown WM, Boore JL: **The complete mitochondrial genome of the articulate brachiopod *Terebratalia transversa***. *Mol Biol Evol* 2001, **18**(9):1734-1744.

13. Serb JM, Lydeard C: **Complete mtDNA sequence of the North American freshwater mussel, *Lampsilis ornata* (Unionidae): an examination of the evolution and phylogenetic utility of mitochondrial genome organization in Bivalvia (Mollusca)**. *Mol Biol Evol* 2003, **20**(11):1854-1866.

14. Boore JL, Brown WM: **Complete DNA sequence of the mitochondrial genome of the black chiton, *Katharina tunicata***. *Genetics* 1994, **138**(2):423-443.

15. Maynard BT, Kerr LJ, McKiernan JM, Jansen ES, Hanna PJ: **Mitochondrial DNA sequence and gene organization in the [corrected] Australian blacklip [corrected] abalone *Haliotis rubra* (leach)**. *Mar Biotechnol (NY)* 2005, **7**(6):645-658.

16. Boore JL: **The complete sequence of the mitochondrial genome of *Nautilus macromphalus* (Mollusca: Cephalopoda)**. *BMC Genomics* 2006, **7**:182.

17. Dreyer H, Steiner G: **The complete sequences and gene organisation of the mitochondrial genomes of the heterodont bivalves *Acanthocardia tuberculata* and *Hiatella arctica* - and the first record for a putative Atpase *subunit 8* gene in marine bivalves**. *Front Zool* 2006, **3**:13-26.

18. Boore JL, Medina M, Rosenberg LA: **Complete sequences of the highly rearranged molluscan mitochondrial genomes of the Scaphopod *Graptacme eborea* and the Bivalve *Mytilus edulis***. *Mol Biol Evol* 2004, **21**(8):1492-1503.

19. Lewis DL, Farr CL, Farquhar AL, Kaguni LS: **Sequence, organization, and evolution of the A+T region of *Drosophila melanogaster* mitochondrial DNA**. *Mol Biol Evol* 1994, **11**(3):523-538.

20. Wilson K, Cahill V, Ballment E, Benzie J: **The complete sequence of the mitochondrial genome of the crustacean *Penaeus monodon*: are malacostracan crustaceans more closely related to insects than to branchiopods?** *Mol Biol Evol* 2000, **17**(6):863-874.

21. Black WCt, Roehrdanz RL: **Mitochondrial gene order is not conserved in arthropods: prostriate and metastriate tick mitochondrial genomes**. *Mol Biol Evol* 1998, **15**(12):1772-1785.

22. Lavrov DV, Brown WM, Boore JL: **A novel type of RNA editing occurs in the mitochondrial tRNAs of the centipede *Lithobius forficatus***. *Proc Natl Acad Sci U S A* 2000, **97**(25):13738-13742.

23. Lavrov DV, Forget L, Kelly M, Lang BF: **Mitochondrial genomes of two demosponges provide insights into an early stage of animal evolution**. *Mol Biol Evol* 2005, **22**(5):1231-1239.

24. Beagley CT, Okimoto R, Wolstenholme DR: **The mitochondrial genome of the sea anemone *Metridium senile* (Cnidaria): introns, a paucity of tRNA genes, and a near-standard genetic code**. *Genetics* 1998, **148**(3):1091-1108.

25. Dellaporta SL, Xu A, Sagasser S, Jakob W, Moreno MA, Buss LW, Schierwater B: **Mitochondrial genome of *Trichoplax adhaerens* supports placozoa as the basal lower metazoan phylum**. *Proc Natl Acad Sci USA* 2006, **103**(23):8751-8756.
